# Course of serum amyloid A (SAA) plasma concentrations in horses undergoing surgery for injuries penetrating synovial structures, an observational clinical study

**DOI:** 10.1186/s12917-017-1057-9

**Published:** 2017-05-22

**Authors:** Eva Haltmayer, Ilse Schwendenwein, Theresia F. Licka

**Affiliations:** 1 Department of Small Animals and Horses, Clinic for Horses, Equine Surgery, University of Veterinary Medicine Vienna, Veterinärplatz 1, A-1210 Vienna, Austria; 2 Department of Pathobiology, Clinical Pathology, University of Veterinary Medicine Vienna, Veterinärplatz 1, A-1210 Vienna, Austria; 3 Department of Veterinary Clinical Studies, Royal (Dick) School of Veterinary Studies, University of Edinburgh, Easter Bush Campus, Midlothian, EH25 9RG Scotland

**Keywords:** Equids, Serum amyloid A (SAA), Injury synovial structure, Septic synovitis, Wound infection

## Abstract

**Background:**

Injuries penetrating synovial structures are common in equine practice and often result in septic synovitis. Significantly increased plasma levels of serum amyloid A (SAA) have been found in various infectious conditions in horses including wounds and septic arthritis. Plasma SAA levels were found to decrease rapidly once the infectious stimulus was eliminated. The purpose of the current study was to investigate the usefulness of serial measurements of plasma SAA as a monitoring tool for the response to treatment of horses presented with injuries penetrating synovial structures. In the current study plasma SAA concentrations were measured every 48 hours (h) during the course of treatment.

**Results:**

A total of 19 horses with a wound penetrating a synovial structure were included in the current study. Horses in Group 1 (*n* = 12) (injuries older than 24 h) only needed one surgical intervention. Patients in this group had significantly lower median plasma SAA levels (*P* = 0.001) between 48 h (median 776 mg/L) and 96 h (median 202 mg/L) after surgery. A significant decrease (*P* = 0.004) in plasma SAA levels was also observed between 96 h after surgery (median 270 mg/L) and 6 days (d) after surgery (median 3 mg/L). Four horses (Group 2) required more than one surgical intervention. In contrast to Group 1 patients in Group 2 had either very high initial plasma concentrations (3378 mg/L), an increase or persistently high concentrations of plasma SAA after the first surgery (median 2525 mg/L). A small group of patients (*n* = 3) (Group 3) were admitted less than 24 h after sustaining a wound. In this group low SAA values at admission (median 23 mg/L) and peak concentrations at 48 h after surgery (median 1016 mg/L) were observed followed by a decrease in plasma SAA concentration over time.

**Conclusions:**

A decrease in plasma SAA concentrations between two consecutive time points could be associated with positive response to treatment in the current study. Therefore, serial measurements of plasma SAA could potentially be used as an additional inexpensive, quick and easy tool for monitoring the treatment response in otherwise healthy horses presented with injuries penetrating synovial structures. However further studies will be necessary to ascertain its clinical utility.

## Background

Injuries penetrating synovial structures are common in equine practice [1, 2, 3]. These injuries cause contamination of the affected synovial structure and can subsequently lead to septic synovitis. Septic synovitis is a serious and potentially lethal condition in horses that requires immediate diagnosis and subsequent treatment [4–6]. Arthroscopic lavage is the recommended treatment of choice [2, 7–9]. However, more invasive approaches have been described in severely affected cases [10–12]. With adequate treatment, survival rates in adult horses range from 84% [6] to 90% [2] with 54% [6] to 81% [2] of horses returning to previous levels of performance [2, 4–6], except horses where solar foot penetration caused septic synovitis. In one larger scale study by Findley et al. [3] survival to discharge was only 56 and 36% returned to their previous athletic function. Introduction of pathogens into a synovial cavity causes a strong inflammatory response resulting in local swelling, heat and pain and eventually leading to enzymatic breakdown of hyaluronic acid and cartilage [7]. The inflammatory response is mainly driven by the release of cytokines from macrophages and monocytes at the site of injury. These pro-inflammatory mediators especially interleukin-1 (IL-1), tumor necrosis factor alpha (TNF-α) and interleukin-6 (IL-6) stimulate the hepatic acute phase protein synthesis [13] once released into circulation. In horses, serum amyloid A (SAA) was shown to be a reliable marker of various septic and non-septic inflammatory conditions [13–18]. SAA is a major acute phase protein in equids and characterized by a rapid and remarkable increase (up to several 1000-fold) from low to undetectable baseline values in plasma concentration and a rapid decrease, once inflammatory or infectious stimuli are eliminated [13, 18]. SAA reaches peak concentrations in plasma about 48 h after the onset of inflammation [18]. Jacobsen et al. [17] were the first to document that septic arthritis elicits a prominent acute phase response with significantly higher SAA concentrations in serum compared to healthy horses and horses with non-septic arthritis. Diagnosis and monitoring of successful treatment in patients with injuries penetrating synovial structures commonly relies on repeated synoviocentesis of the affected structure and subsequent synovial fluid analysis [19] as well as clinical signs such as lameness, heat and swelling. Synovial fluid analysis can be inconclusive under certain circumstances especially after repeated synoviocenteses [17, 20, 21] or drug application [20, 21]. Moreover, repeated synoviocentesis and subsequent collection of synovial samples can be difficult, especially under field conditions. Fibrin formation, synovial hypertrophy, and loss of synovial fluid via drainage through an open wound can also interfere with successful collection of a synovial fluid sample. To our knowledge, there are only limited reports about serial SAA measurements [17] over the course of treatment for assessment of treatment success.

The aim of the present study was to evaluate the course of plasma SAA over time in patients undergoing treatment for injuries penetrating synovial structures and to evaluate plasma SAA as a potential marker for treatment response. Hypothesis tested is that plasma SAA concentrations decrease significantly in response to successful treatment.

## Methods

### Animals

Horses over 1 year of age admitted to the Equine Hospital, University of Veterinary Medicine Vienna, Austria for investigation and treatment of injuries penetrating synovial structures between May 2012 and January 2013 were included in the study. Inclusion criteria were injuries (lacerations, open fractures, puncture wounds and street nail injury) penetrating synovial structures and requiring surgical intervention. Pregnant mares and horses younger than 1 year, as well as horses with clinically relevant medical conditions prior to the development of the presenting complaint such as horses with additional, not wound associated infections or inflammatory conditions were excluded from the study.

Remnants of plasma samples obtained for routine blood work were used for SAA measurements, or blood was withdrawn from a previously placed intravenous (IV) catheter. No invasive procedure was performed for study purposes only. Owner’s written consent was obtained at admission.

For description of results and statistical analysis horses were divided into three Groups. Group 1 (*n* = 12) consisted of horses with injuries older than 24 h, Group 2 (*n* = 4) were horses that required more than one surgical intervention/one lavage and Group 3 (*n* = 3) consisted of horses with fresh (< 24 h) injuries. Decision for further lavages was taken by the clinician in charge of the case based on clinical findings and results of synovial fluid analysis.

### Initial assessment

A thorough clinical examination and routine blood work (complete blood count, creatinine, total protein, glutamate dehydrogenase, gamma-glutamyl transferase, creatinine kinase, potassium and fibrinogen) was performed on each patient. Samples for routine blood work were obtained by venipuncture of the jugular vein using a vacutainer system (Vacuette, Greiner Bio-One GmbH, Frickenhausen, Germany) or from a venous catheter. On admission, horses underwent a limited orthopaedic examination, as was decided relevant by the clinician in charge of the case. No evaluation at trot, no flexion tests and no diagnostic anaesthesia was performed, as the horses were lame at the walk and/or had obvious wounds and swellings. Additionally, radiographic and/or ultrasonographic examinations were performed.

Centesis of at least 1 synovial structure adjacent to the injury was carried out, and when possible synovial fluid was collected. If no synovial fluid sample could be obtained, the involved synovial structure was distended with sterile saline solution to identify communication with a wound. In cases where no synovial fluid sample could be aspirated sterile saline solution was injected and immediately re-aspirated to obtain a synovial lavage sample. Synovial samples were analysed at the Clinical Pathology Unit, University of Veterinary Medicine Vienna, Austria by one of the authors (IS). A smear and a cytospin were prepared from native and lavage samples and stained with a Romanowsky-type stain (Haemafix™ Biomed Labordiagnostik GmbH, Oberschleißheim, Germany) for verification of automated cell counts, cell differentiation and evaluation of mucin precipitate. The concentration of nucleated cells (TNCC) was determined in undiluted samples with a laser based hematology system (Advia 2120™, Siemens Diagnostics, Erlangen, Germany). To samples with high viscosity, which were inappropriate for automated counting a grain of hyaluronidase powder was added, so that they could be analysed. In diluted samples only the ratio of neutrophils and monocytes was analysed. Total protein concentration was evaluated by refractometry from undiluted samples. An injury penetrating synovial structures was confirmed when communication of the injury with a synovial cavity was found (positive pressure test) and/or synovial fluid analysis results showed TNCC >20 × 10^3^/μL and/or neutrophils >80% and total protein >4 g/dL [19, 22]. Pathological findings on physical examination (lameness, swelling, heat, fever) were also taken into account.

### Preoperative treatment and surgery

All treatment decisions were carried out by the clinician in charge of the case. Preoperatively, an intravenous catheter was placed in a jugular vein, and all patients received systemic broad spectrum antimicrobials: penicillin (30.000 IU/kg, IV, every 6 h) and gentamicin (6.6 mg/kg, IV, once daily). When indicated by results of antimicrobial susceptibility tests, marbofloxacin (2 mg/kg, IV, once daily) or cefquinome (2.2 mg/kg, IV, once daily) were used. All patients received non-steroidal anti-inflammatory drugs (NSAIDs): flunixin meglumine (1.1 mg/kg, IV, twice daily) or phenylbutazone (2 mg/kg, PO, twice daily). Patients were treated surgically by standard wound debridement and lavage of the affected synovial structure under general anaesthesia or standing under sedation and regional anaesthesia. Lavage was performed either through 16G and 18G needles or arthroscopically. All affected synovial structures were closed after lavaging. Details about patients and treatment procedures are displayed in Table 1.

**Table 1:** Detailed patient information reporting age (in years), breed, sex, diagnosis, treatment, complications and synovial fluid parameters

Group	Patient	Signalement	Diagnosis	Surgical treatment	Repeated AL/standing lavage	Complications	Duration of injury	Synovial parameters at admission^a^	Duration of treatment
1	2	Trotter, 6y, S	Pastern laceration right hind, septic digital flexor tendon sheath	Wound debridement; standing needle lavage digital flexor tendon sheath		None	>24 h	Positive pressure test; >80% neutrophils; 3200 TNC/μL; TP 4 g/dL	4 days treatment was continued by referring veterinarian
1	5	Gidran, 13y, G	Laceration left tarsus, septic tarsal sheath	Wound debridement; AL tarsal sheath; GA		None	2 days	>85% neutrophils; and positive pressure test	9 days
1	6	Cob, 13y, M	Street nail injury right front, septic distal interphalangeal joint	AL distal interphalangeal joint; GA		Euthanasia d 30 of hospitalization after developing septic navicular bursitis right front	2 days	>95% neutrophils; 200 TNC /400xHPF; TP 7,2 g/dL	15 days for septic interphalangeal joint
1	8	Nonius, 9y, G	Street nail injury left hind, septic navicular bursa	BL navicluar bursa:; GA		none	5 days	>95% neutrophils; positive pressure test	14 days
1	11	WB, 18y, G	Puncture wound left hind fetlock, cellulitis	Debridement and standing needle lavage metatarsophalangeal joint		Euthanasia after discharge due to laminitis right hind	>2 days	80% neutrophils; 3200 TNC/μL; TP 3 g/dL	16 days
1	12	Cob, 16y, M	Laceration left tarsus septic tarsometatarsal joint	needle lavage tarsometatarsal joint: GA		Euthanasia on d 27 of hospitalisation due to osteomyelitis left Mt. IV	4 days	>95% neutrophils; positive pressure test	19 days for septic tarsometatarsal joint
1	13	Pony, 20y, G	Laceration, bone sequester left humerus, septic bicipital bursa	Needle lavage, sequestrectomy, standing		None	>24 h	Positive pressure test	28 days
1	14	Icelandic horse, 15y, M	Laceration left elbow; septic elbow joint, open intraarticular olecranon fracture Type II	AL elbow joint, fracture repair; GA		None	>24 h	Positive pressure test	26 days
1	15	WB, 24y, G	Laceration SDFT and DDFT right hind, septic digital flexor tendon sheath	TVL digital flexor tendon sheath; GA		Scar tissue formation SDFT	2 days	Positive pressure test; >75% neutrophils; 21,910 TNC/μL; TP 4 g/dL	10 days
1	16	Icelandic horse, 2y, M	Laceration right carpus septic carpal joints	AL carpal joints; GA		None	>36 h	Positive pressure test; >80% neutrophils; 26,670 TNC/μL; TP 3,4 g/dL	12 days
1	18	Arab, 17y, M	Laceration left tarsus, septic tarsocrural joint/open comminuted calcaneus fracture	standing needle lavage tarsocrural joint;		Euthanasia on d 7 of hospitalisation due to pleural effusion, heart failure	>36 h	Positive pressure test; >96% neutrophils; 10,270 TNC/μL; TP 5 g/dL	6 days
1	20	WB, 12y, G	Laceration right front fetlock septic digital flexor tendon sheath	TVL digital flexor tendon sheath; GA		Adhesions formation SDFT and digital tendon sheath;	7 days	Positive pressure test; >88% neutrophils; 310 TNC/400× HPF; TP 3,5 g/dL	18 days
2	4	WB, 8y, G	Laceration right stifle; septic femoropatellar joint	Wound debridement; AL femoropatellar joint: GA	Yes; AL; GA; 48 h after first surgery	None	36 h	Positive pressure test; >80% neutrophils; 8700 TNC/μL; TP 4,6 g/dL	9 days
2	7	QH, 4y, G	Laceration right carpus septic middle carpal joint; osteomyelitis carpal accessory bone	AL middle carpal joint: GA	8 days after first surgery; curettage of carpal accessory bone, GA; no AL indicated	Osteomyelitis accessory carpal bone	>2 days	positive pressure test; >80% neutrophils; 120 TNC/400× HPF; TP 6,4 g/dL	28 days
2	17	Polo Pony, 16y, G	Puncture wound left tarsus; septic tarsal sheath	AL tarsal sheath; GA	Yes; AL; GA 96 h after initial surgery		4 days	>95% neutrophils; >100 TNC/400× HPF; TP 7,2 g/dL	23 days
2	19	Trotter, 8y, M	Laceration right carpus septic carpal joints	AL carpal joints; GA	Yes; 3 ALs GA 48 h, 96 h and 6 days after initial surgery		5 days	>90% neutrophils; 36,600 TNC/μL; TP 6 g/dL	36 days
3	1	WB, 3y, M	Laceration left tarsus, contaminated tarsocrural joint	wound debridement; AL tarsocrural joint: GA		None	5 h	>90% neutrophils; 35,880 TNC/μL; TP 5 g/dL	12 days
3	3	Criollo, 20y, M	Street nail injury right hind; contaminated navicular bursa	BL navicular bursa; GA		None	6 h	Positive pressure test	20 days
3	9	WB, 9y, M	Pastern laceration left hind, contaminated digital flexor tendon sheath	TVL digital flexor tendon sheath; GA		None	4 h	Positive pressure test; >95% neutrophils; 14,220 TNC/μL; TP 2,4 g/dL	10 days

*M* mare, *S* stallion, *G* gelding, *WB* warmblood, *QH* quarter horse, *Mt. IV* fourth metatarsal bone, *DDFT* deep digital flexor tendon, *SDFT* superficial digital flexor tendon, *GA* general anaesthesia, *DRLP* distal regional limb perfusion, *AL* arthroscopic lavage, *TVL* tendovaginoscopic lavage, *BL* bursoscopic lavage, *HPF* high power field, *TP* total protein

^a^not all parameters were available for each patient, dependent on the amount of synovial fluid/if synovial fluid was obtained

### Postoperative management

After surgery horses were kept on systemic antimicrobials and NSAIDs. Antimicrobials, a combination of 250 IU bacitracin and 5000 IU neomycin or 500 mg amikacin were instilled into the synovial structure after lavage and after each synovial centesis. In some cases distal regional limb perfusions (DRLP) with 500 mg amikacin were performed in addition. During hospitalization, horses were examined clinically at least once a day (lameness assessment at the walk and physical examination). Horses received bandage changes every 2–3 days or as needed.

### SAA samples and assay

Blood samples for plasma SAA measurements were taken at admission (before initial surgical treatment) and subsequently every 48 h until infection was considered to be resolved (improved clinical presentation, synovial fluid analysis within normal limits)-this was taken as a favourable response to treatment. Samples were collected from a previously placed intravenous catheter or where routine blood work was taken at time point representing the 48 h interval remnants of plasma samples from this routine blood work were used. Venous blood samples were collected into blood tubes containing heparin and centrifuged. The supernatant was either analysed immediately, if the clinician in charge of the case decided to include SAA analysis in the routine blood work, or frozen at −20 °C and stored for a maximum of 60 days until analysis. Analyses were performed at the Clinical Pathology Unit, University of Veterinary Medicine Vienna, Austria, using a commercially available immunoturbidimetric assay (LZ test SAA, Eiken Chemical Co, Tokyo, Japan) validated for use in horses in a prior study [23] and re-evaluated and adapted on a fully selective autoanalyser for clinical chemistry (Cobas ™501c, Roche Diagnostics, Vienna, Austria) by the Department for Clinical Pathology [24]. Cut-off value of <10 mg/L was established for the local population of healthy horses prior to the present study [24].

### Statistical methods

Patients of Group 2 and Group 3 were excluded from statistical analysis due to the low number of patients.

Statistical analyses were performed using MedCalc 11.3.1.0 package (MedCalc Software bvba, Ostend, Belgium). All probabilities were two-tailed and *P* values <0.05 were regarded significant. To test for normality a Kolmogorov-Smirnov test was performed. Univariate comparisons of two consecutive plasma SAA measurements were performed with the non-parametric Wilcoxon test for paired samples (respective *P*-values were not adjusted for multiple comparisons).

## Results

### Study population and samples

Nineteen horses admitted to the University Clinic for Horses, University of Veterinary Medicine Vienna, Austria, between May 2012 and January 2013 matched the inclusion criteria. Horses were of mixed breed and age. Females and males were evenly distributed.

Patient details including clinical data and surgical procedures are displayed in Table 1.

In 15 patients an arthroscopy/bursoscopy/tendovaginoscopy and lavage of the affected synovial structure was performed under general anaesthesia.

In 5 patients wound revision and lavage of the affected synovial structure was performed standing under sedation and regional anaesthesia through 16G or 18G needles. Decision on needle lavage was based on the presence of a calcaneal fracture in case 18, on the risk of a humeral fracture during recovery in case 13, on the anatomy of the joint involved in case 12 and on financial considerations in the remaining cases.

Post operatively horses were kept on systemic broad-spectrum antimicrobials and NSAIDs for a median of 10.5 days (range 7–25 days). Four patients received a median of 3 (range 2–4) DRLPs with antimicrobials. In 9 patients repeated synoviocentesis was performed to monitor treatment success and intrasynovial antimicrobials were administered after each procedure.

### Plasma SAA concentrations

Statistical analysis was not performed in Group 2 and 3 due to the limited number of patients.

Median plasma SAA values of all Groups are displayed in Table 2.

**Table 2:** Median (range) of plasma SAA values (mg/L) of horses over their course of treatment for injuries penetrating synovial structures. Group 1: horses with injuries older than 24 h requiring only a single surgical intervention, Group 2: horses that required more than one surgical intervention, Group 3: horses with wounds of less than 24 h

	pre OP	48 h	96 h	6 days^*^
Group 1 (*n* = 12)	454 (275–6378) a	776 (422–2100) a	201 (19–864) b	5 (0–17) c
Group 2 (*n* = 4)	940 (16–3378)	2525 (495–4000)	2593 (811–3370)	1000 (122–2618)
Group 3 (*n* = 3)	23 (7–77)	1016 (273–1666)	297 (159–500)	5 (0–17)

*pre OP* preoperative

Different letters indicate significant difference (*P* < 0.05) between time-points of SAA measurements during treatment

^*^At time point 6 days data was available from 9 horses in Group 1, 4 horses in Group 2 and 2 horses in Group 3.

**Fig. 1:**
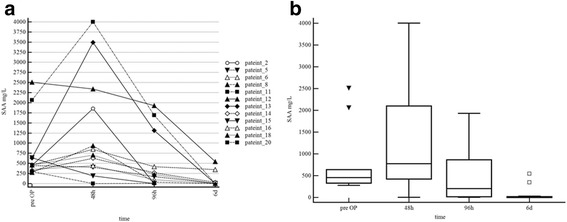
**a** Course of plasma SAA of all patients in Group 2. Peak values could be observed between pre OP (*n* = 4) and 48 h after surgery (*n* = 7). Notice the continuous decrease of plasma SAA concentrations after surgery. **b** *Box* and *whisker plots* of course of plasma SAA concentration during treatment of patients of Group 1 Explanations: In *box-and-whisker plots*, *central box* represents values from lower to upper quartile, *middle line* represents the median; *whiskers* extend from minimum to maximum value, excluding outside and far out values which are displayed as separate points

Patients in Group 1 showed a significant decrease in plasma SAA concentrations over the course of treatment: Patients in that group had two different rise and fall pattern. Four individuals showed the peak concentration at time of admission and the remaining patients had peak concentrations at 48 h after surgery. Time of injury to admission was not connected to one of the rise and fall pattern in Group 1 (Fig. 1a).

The median percentage decrease of plasma SAA in this group was 70% (range 18–100%) between 48 and 96 h and 99% (range 17–100%) between 96 h and 6 days after initial surgical intervention. Results of pairwise comparison of two time points of measurement are displayed in Fig. 1b.

**Fig. 2:**
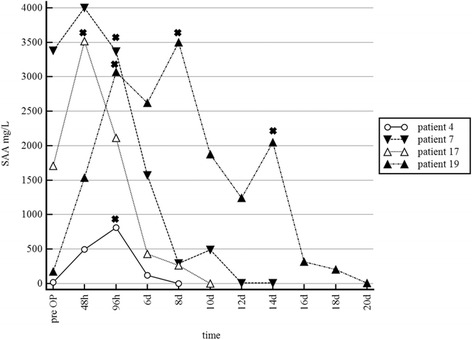
Course of plasma SAA in horses of Group 2 Timepoints of surgery are marked (+). In patients 4 and 19 an increase of plasma SAA was observed before the following surgical treatment. Patient 7 showed very high initial SAA values with only a minor decrease before the second surgical intervention. Patient 17 showed an unusual high increase after the first surgery

Patients in Group 2 showed variable patterns of plasma SAA concentration over time. Two horses (4, 19) showed persistently high or even increased levels of SAA after the first surgery, patient 17 showed a very high increase between the first and the second surgery and patient 7 showed very high initial SAA values and only a minor decrease before the second surgery (Fig. 2). This represents a median increase in plasma SAA concentrations of 24% (range − 40 − +100%) between 48 and 96 h and a median decrease of only 60% (range 15–85%) between 96 h and 6 days after the first surgery. Especially in Patient 19 the increase before surgery can be well observed (Fig. 2).

**Fig. 3:**
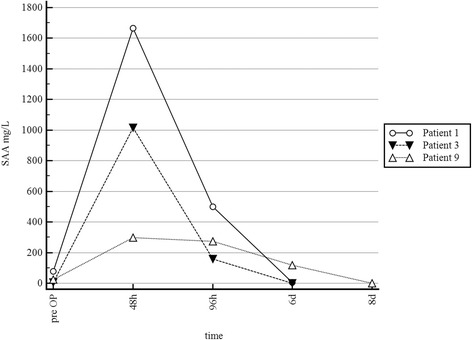
Illustration of the typical rise and fall pattern of plasma SAA concentrations of patients with injuries <24 h. Peak concentrations are observed 48 h after surgery. Notice the relative low pre OP plasma SAA values

Patients in Group 3 showed typical rise and fall pattern with a peak at 48 h after initial surgical treatment. In that Group injuries happened <24 h before admission (Fig. 3). The median decrease of plasma SAA levels was 60% (range 9–84%) between 48 and 9 h post initial treatment and 98% (range 61–100%) between 96 h and 6 days after surgery.

## Discussion

In the current study we monitored the course of plasma SAA concentrations in response to treatment in 19 otherwise healthy horses with injuries penetrating synovial structures. Plasma SAA concentrations in cases with injuries penetrating synovial structures decreased rapidly in response to treatment and returned to levels below the reference value (10 mg/L) during the course of successful treatment (Figs.1a, 3). Therefore SAA analysis offers a timely and useful tool for monitoring the effect of treatment of injuries penetrating synovial structures. Jacobsen et al. [17] mentioned in their study that plasma SAA concentration in one horse with septic arthritis decreased during the course of treatment. This could also be observed in the present study in horses treated for injuries penetrating synovial structures. To the authors’ knowledge, this is the first study investigating the utility of serial plasma SAA measurements for monitoring treatment response in patients with injuries penetrating synovial structures in a clinical set up. Data indicates that the course of plasma SAA concentration reflects the response to treatment well. Patients of Group 1 and 3 with a highly favourable response to initial surgical treatment showed a continuous decrease of SAA concentrations between 48 and 96 h after surgery as well 6 days after surgery. In contrast in cases with ongoing infection (Group 2) different patterns of SAA could be observed. Two horses (4, 19) showed persistently high or even increased levels of SAA after the first surgery, one horse (17) showed a very high increase between the first and the second surgery and the other horse (7) showed very high initial SAA values and only a minor decrease before the second surgery. However this has to be interpreted with care due to the low number of patients and no conclusion can be drawn from the data at this point.

Persistently high concentrations of SAA have been previously reported in horses with complications after elective and emergency surgery [14, 25, 26]. In the present study this was considered to be an indication for lack of treatment response. However, further surgical treatment was instigated only with corresponding clinical signs and synovial fluid analysis and was not based on SAA values. This does not preclude that animals could have potentially improved without additional intervention. However, such an experimental set up cannot be used in client owned horses. The time interval of 48 h for SAA sample collection in the present study was chosen because SAA shows peak concentration 48 h after onset of infection [13, 18]. Concentrations of SAA correspond to the severity of tissue damage as well as to the volume of damaged tissue [18] and drop rapidly as soon as synthesis stops [18]. Due to these unique features of SAA sequential measurements of plasma SAA concentrations were suggested as a useful aid in patient management, planning of treatment strategies and in decision-making whether to perform surgery or not [13, 18].

Duration of the disease process prior to admission as well as the stage of the disease process at admission have to be carefully considered when interpreting SAA concentrations in a patient [17, 18]. In the current study patients in Group 3 had normal or very low SAA concentrations (range from 6.7 mg/L to 23.1 mg/L) at the time of admission, despite a positive pressure test and/or neutrophil counts >90% in synovial fluid. In these patients the reported duration between detection of injury by the owner and hospital admission was <24 h thus below the reported lag time for SAA increase. Jacobsen et al. [17] also described one case where the authors assumed that the time from injury to sample collection was too short for the local inflammatory response to induce hepatic SAA synthesis. In this particular case the delay between injury and sample collection was only 2 h. The three patients in Group 3 support this suggestion [17]. Therefore care has to be taken when interpreting single SAA values, especially in early stages of disease and results of the current study strongly suggest that SAA values obtained at a single time point should not be used for diagnostic purposes. Also low-grade infections [14] and well sequestrated infections [14] were previously reported to cause a retarded response in plasma SAA in relation to clinical signs. This can be explained by a local inflammatory response that is not “strong” enough to trigger SAA synthesis in the liver and incite a systemic inflammatory response. In contrast Belgrave et al. [14] reported that SAA values were increased 24 h prior to onset of clinical signs in horses with colic and metritis [13] and therefore SAA could potentially aid in early detection of inflammation. However due to the limited number of patients in Group 3 no conclusions can be drawn at this point.

In the present study most patients showed peak concentration of plasma SAA 48 h after surgery. In all cases surgery was performed on the day of hospital admission. Therefore, peak values might have been influenced by the degree of infection of the injury site and/or the synovial structure, as well as by surgical trauma [25–27], general anaesthesia [28, 29] or antimicrobial treatment [30]. Jacobsen et al. [27] reported slightly increased plasma SAA values up to 5 days after arthroscopy (up to 100 mg/L), however these levels remain much lower than in the cases of the present study [26]. Sanchez-Teran et al. [31, 32] recently suggested in their study that in healthy adult horses neither arthroscopic lavage nor through-and-through joint lavage with needles influenced plasma SAA concentrations 48–120 h after surgery. Moreover, Pollock et al. [26] described that healthy horses undergoing an elective surgery responded similar to surgical trauma than horses with elevated plasma SAA concentrations undergoing emergency surgery. In four patients in Group 1 a decrease in plasma SAA could be observed between admission and 48 h after surgery (Fig. 1a). These results do not support the suggestion that surgical trauma alone led to the typical rise and fall pattern post-surgery, however due to the low number of cases no conclusion can be drawn.

Currently little and controversial information is available regarding the effects of general anaesthesia on plasma SAA concentrations. One study reported no increase in plasma SAA concentrations after general anaesthesia [28], another study reported values of up to 520.7 mg/l in horses 24 h after general anaesthesia of a maximum of 2 h duration without surgery [29]. However, the latter study also reported high inter-individual differences so that high and low responders among horses could be discussed.

All patients enrolled in the current study received NSAIDs during the course of treatment. The effect of non-steroidal anti-inflammatory drugs on the acute phase response has been investigated studies in various species (dogs, ruminants, humans) other than horses [33–35]. These studies showed that administration of non-steroidal anti-inflammatory drugs did not affect the acute phase response in comparison to a non-treated control group. On the other hand some studies exist that state the opposite [36, 37]. Based on these contradictory information it remains unclear whether the course of SAA was affected by administration of NSAIDs in horses in the current study. This leaves room for further studies investigating the effect of NSAIDs on SAA response in horses with injuries penetrating synovial structures. Increased plasma SAA concentrations were observed in pregnant mares starting 1 week before parturition until 1 month post partum [16]. Therefore pregnant mares were excluded from the present study. Horses younger than 1 year were also excluded from the present study because differentiation between traumatic septic arthritis and septic arthritis/physitis in foals was deemed to be potentially imprecise.

Nunokawa et al. [38] described in their study that various inflammatory and infectious diseases lead to increased plasma SAA concentrations in horses. Therefore in patients with multiple inflammatory processes plasma SAA concentrations are only of limited use for monitoring response to treatment for injuries penetrating synovial structures. In horses with postoperative complications unrelated to the site of injury that reportedly lead to an increase in plasma SAA concentration such as gastrointestinal pathologies [39, 40] (eg. colitis) interpretation of SAA concentrations regarding the state of infection of the primary injury by a single measurement might be challenging or even impossible.

One limitation of the present study is that patients presented with a variety of wounds and different types of tissues involved. This, however, reflects a typical hospital population and therefore allows testing the hypothesis in a routine clinical setting.

These wounds led to various amounts of tissue damage and infection of tissue surrounding the synovial structure. Therefore it stands to reason that the pattern of SAA during the course of treatment was likewise influenced by resolution of infection from the wound and/or synovial structure.

In the present study two patients had fractures in addition to the injuries penetrating synovial structures. Plasma SAA concentrations therefore may have been influenced by the surgical trauma due to fracture repair in patient 14 and trauma to the bone itself in both patients. In patient 14 serial SAA measurements showed a decrease over the course of treatment which was considered a reflection of favourable response to treatment. Patient 18 was euthanized due to heart failure. However synovial analysis prior to euthanasia revealed no synovial sepsis. In Patient 12 osteomyeltis of the fourth metatarsal bone was diagnosed after synovial sepsis of the tarsometatarsal joint was considered to be improved (clinical improvement, low SAA). Consent for further treatment was not given by the owner and the patient was euthanised. In this case plasma SAA was within normal limits despite the presence of osteomyelitis. The reason for the lack of increased plasma SAA could not be determined in this case. Another limitation of the present study is, that due to the clinical design, the number of plasma samples obtained per horse could not be standardised more stringently. Number of samples obtained depended on the time required for resolution of infection.

## Conclusions

In summary the results of the present study suggest that sequential measurement of SAA exhibiting continuously declining concentrations seems to indicate a favourable response to treatment. Serial plasma SAA concentrations potentially offer a relatively inexpensive, non-invasive and rapid method to monitor response to treatment in injuries penetrating synovial structures. However due to the small number of patients and the variation in the data of the current study further investigations will be necessary to ascertain the clinical value of plasma SAA as a complementary monitoring tool in horses with injuries penetrating synovial structures.

## Abbreviations

AL: Arthroscopic lavage
BL: Bursoscopic lavage
d: Day (s)
DDFT: Deep digital flexor tendon
DRLP: Distal regional limb perfusion
G: Gelding
GA: General anaesthesia
h: Hour (s)
HPF: High power field
IL**-**1: Interleukin-1
IL**-**6: Interleukin-6
IQR: Interquartile range
IV: Intravenous
M: Mare
Mt. IV: Fourth metatarsal bone
NSIAD: Non steroidal anti-inflammatory drug
preOP: Preoperative
QH: Quarter horse
S: Stallion
SAA: Serum amyloid A
SDFT: Superficial digital flexor tendon
TMT: Tarsometatarsal joint
TNC: Total nucleated cells
TNCC: Total nucleated cell count
TNF**-**α: Tumor necrosis factor alpha
TP: Total protein
TVL: Tendovaginoscopic lavage
WB: Warmblood
